# Stochastic resonance at criticality in a network model of the human cortex

**DOI:** 10.1038/s41598-017-13400-5

**Published:** 2017-10-12

**Authors:** Bertha Vázquez-Rodríguez, Andrea Avena-Koenigsberger, Olaf Sporns, Alessandra Griffa, Patric Hagmann, Hernán Larralde

**Affiliations:** 1 Universidad Nacional Autónoma de México, Instituto de Ciencias Físicas, Cuernavaca, Mexico; 2 Indiana University, Department of Psychological and Brain Sciences, Bloomington IN, USA; 3 Lausanne University Hospital (CHUV), Department of Radiology, Lausanne, Switzerland; 4 University of Lausanne (UNIL), Lausanne, Switzerland

## Abstract

Stochastic resonance is a phenomenon in which noise enhances the response of a system to an input signal. The brain is an example of a system that has to detect and transmit signals in a noisy environment, suggesting that it is a good candidate to take advantage of stochastic resonance. In this work, we aim to identify the optimal levels of noise that promote signal transmission through a simple network model of the human brain. Specifically, using a dynamic model implemented on an anatomical brain network (connectome), we investigate the similarity between an input signal and a signal that has traveled across the network while the system is subject to different noise levels. We find that non-zero levels of noise enhance the similarity between the input signal and the signal that has traveled through the system. The optimal noise level is not unique; rather, there is a set of parameter values at which the information is transmitted with greater precision, this set corresponds to the parameter values that place the system in a critical regime. The multiplicity of critical points in our model allows it to adapt to different noise situations and remain at criticality.

## Introduction

Random noise has been traditionally considered as an obstacle in the transmission of information, contaminating accurate communication and limiting the achievable information rate^1,2^. Nonetheless there are examples in which the presence of noise makes substantial improvements in signal detection^3–5^, through the phenomenon of stochastic resonance (SR).

SR was proposed as a possible explanation for the periodicity of the ice ages on Earth^6^, and has been studied in Schmitt triggers^7^, tunnel diodes^8^ and bidirectional ring lasers^9^. Moreover, it was shown that stochastic noise plays a role in neuroautonomic regulation of the heart rate to generate complex dynamics like variability and scale invariance across a range of scales that are a hallmark of criticality^10^. Nowadays, the effects of noise on biological sensory systems is being extensively explored. One of the first demonstrations of SR in the nervous system was carried out on crayfish mechanoreceptors^3,11^. Since then, other experimental demonstrations have included neurons in crickets^12^, rats^13,14^, and cats^5^, along with several studies in humans on the enhancement of detection and transmission in the sensorimotor system during a motor task^4,15^. In^16^ the propagation of a periodic input signal through an Erdös-Rényi network for different noise levels was studied, and it was found that noise indeed enhanced signal propagation in the model. However, to our knowledge, no studies have explored the SR phenomenon as a mechanism that could potentially enhance the transmission of information along axonal pathways in the human brain.

Growing evidence supports the hypothesis that the dynamics of the brain resembles the dynamics of a system near a critical point. This suggests that many functionally important features of brain dynamics may be optimized at criticality^17–26^? Recent work has shown that a discrete state dynamical model implemented on a network of neuroanatomical connections (connectome^27^) exhibits a phase transition similar to that observed in a percolation model, where the average size of the second biggest cluster of active nodes reaches its maximum value for a specific activation threshold^28^. Furthermore, the model presented in^28^ is capable of replicating spontaneous brain activity patterns that resemble so-called resting state networks^29^, which are widely regarded as key components of functional brain architecture^30^. Other experiments have demonstrated that the dynamic range (the range of stimulus intensities that allows network responses to be distinguished)^31^, mutual information and information capacity appear to be maximized at critical points^32,33^.

It is not clear, however, whether the levels of noise that increase the transmission of information through the brain are related to criticality. This issue is important because the brain, even when noise sources are present, must be capable of integrating information across multiple sensory modalities and brain systems, in order to generate adaptive neural and behavioral responses^2,17^. In the present work we propose a simple integrate and fire discrete dynamic model^16,28^ in which nodes can activate spontaneously^34–36^. This spontaneous activation acts as a noise. In this work we determine quantitatively the amount of noise required for the best transmission of signals through the structural network of the brain’s connectome, and its relationship with the hypothesis of the brain operating near criticality^17,18,20,24–26,32^.

## Results

### The model

The model is implemented on a network representing a human connectome. Each node in this network represents a gray matter region of the human cortex whereas edges represent white matter fiber tracts that connect cortical regions. The weighted elements (*w*_*ij*_) of the adjacency matrix of the connectome are proportional to the number of streamlines connecting two brain regions, indicating the strength of a connection between nodes *i* and *j*. The method used to obtain the weighted network matrix of the human connectome is reported in^37^ and is briefly described in the Supplementary Material section.

The network contains *N* = 114 nodes with binary states that are updated synchronously according to a dynamic rule adapted from^28^. Each node, characterized by a boolean variable *s*_*i*_, is updated every time step and can be in one of two states: quiescent *Q* (with *s*_*i*_ = 0) or excited *E* (with *s*_*i*_ = 1). The state of each node obeys the following transition rules:

- *Q* → *E* with a probability *P*_*QE*_ (corresponding to spontaneous activation of the node) or if the input signal ${\alpha }_{i}=\sum _{j=0}^{{k}_{i}}{w}_{ij}{s}_{j}(t)$ is higher than a threshold *T*.
- *E* → *E* with a probability *P*_*EE*_ if the node was still stimulated to be activated as above, i.e. with a probability *P*_*QE*_ or if *α*_*i*_ > *T*.
- *E* → *Q* with a probability (1 − *P*_*EE*_) or with a probability *P*_*EE*_ provided that the stimulus received is not large enough to maintain the node active.

The probability *P*_*QE*_, i.e. the probability *that a node activates spontaneously* ^34,35^, *plays* the role of the noise in the system, thus *P*_*QE*_ is the quantity that we expect to be connected with SR^2,24^. *P*_*EE*_ represents the probability that the node has enough material/energy to fire for more than one time step (as may be the case if some of the neurons in that brain region have not fired yet). It is important to highlight that there is no refractory state in the model because the nodes represent whole brain regions comprising large populations of neurons, not individual nerve cells.

Thus the state of the i-th node changes in time according to the following dynamical rule:

1 $$\begin{array}{rcl}{s}_{i}(t+\mathrm{1)} & = & \{1+{s}_{i}(t)[H({P}_{EE}-{r}_{2})-\mathrm{1]}\}\\  &  & \times \{H({P}_{QE}-{r}_{1})+[1-H({P}_{QE}-{r}_{1})]H({\alpha }_{i}-T)\}\end{array}$$

where *r*_1_ and *r*_2_ are independent random numbers drawn from a uniform distribution between zero and one; and *H*(*x*) is a step function (with *H*(*x*) = 1 if *x* ≥ 0 and *H*(*x*) = 0 otherwise).

### Measuring the transmission of signals through the network

The SR we envision in this paper is along the lines of that described in refs^38,39^, where a signal is detected if it is stronger than a certain threshold. Thus, a weak signal, by itself, will not be observed. When noise is added, the signal plus noise may cross the threshold allowing the signal to be detected. However, if there is too much noise, we lose all the information about the signal. Hence, there must be a level of noise to be added to a weak signal that optimizes the detection of the signal.

To evaluate the transmission of information through the network, we introduced a signal in the system, one node at a time. This signal consisted in switching the node’s activity to be in the excited (*E*) state for a certain number of time steps and then in the quiescent (*Q*) state for the same number of steps (these results were obtained for an input signal of a frequency of 1/50, but we have obtained the same results for frequencies of up to 1/200); this pattern was continued periodically throughout the evolution of the system. While the input signal was being delivered through a specific input node *i*, we let the rest of the system evolve according to the dynamic rule and evaluate the *similarity* between the input signal and the output signal -the activity- at each node.

We use the Fourier spectrum of the signals to measure their *similarity*. If the difference between two signals is that they are merely rescaled or shifted, their power spectrum will have the same principal frequencies, but not the same amplitudes, yet, we would want to say that the signals are *similar*. Accordingly, we search for a factor (*λ*_*ij*_) that will minimize the weighted squared difference between the input and output spectrum. To determine this factor, each term of the squared difference is multiplied by the amplitude of the input spectrum, so the frequencies with the larger amplitudes will have a greater contribution in the sum, and hence, in the determination of *λ*_*ij*_. Finally, we define the similarity between the signals as:

2 $$sim(i,j)=-\mathrm{log}(\frac{1}{\sum _{m}^{M}{({\varphi }_{m}^{i})}^{3}}\sum _{n}^{M}{({\varphi }_{n}^{i}-{\lambda }_{ij}{\varphi }_{n}^{j})}^{2}{\varphi }_{n}^{i}),$$

where

3 $${\lambda }_{ij}=\frac{{\sum }_{m}^{M}{({\varphi }_{m}^{i})}^{2}{\varphi }_{m}^{j}}{{\sum }_{m}^{M}{({\varphi }_{m}^{j})}^{2}{\varphi }_{m}^{i}}$$

and ${\varphi }_{n}^{i}$ is the amplitude of the n-th main frequency of the signal input in node *i* (input node, or “seeder”), and ${\varphi }_{n}^{j}$ the amplitude for the same frequency in the power spectrum of the activity at node *j* (output node, or “receiver”). To compute this quantity we use only the *M* principal frequencies of the node *i*, that are the ones that have an amplitude larger than 0.0001. The normalization factor allows us to compare this measure for different input signals and the −*log* will make *sim*(*i*, *j*) maximum when the difference between the two spectra is the lowest.

**Figure 1:**
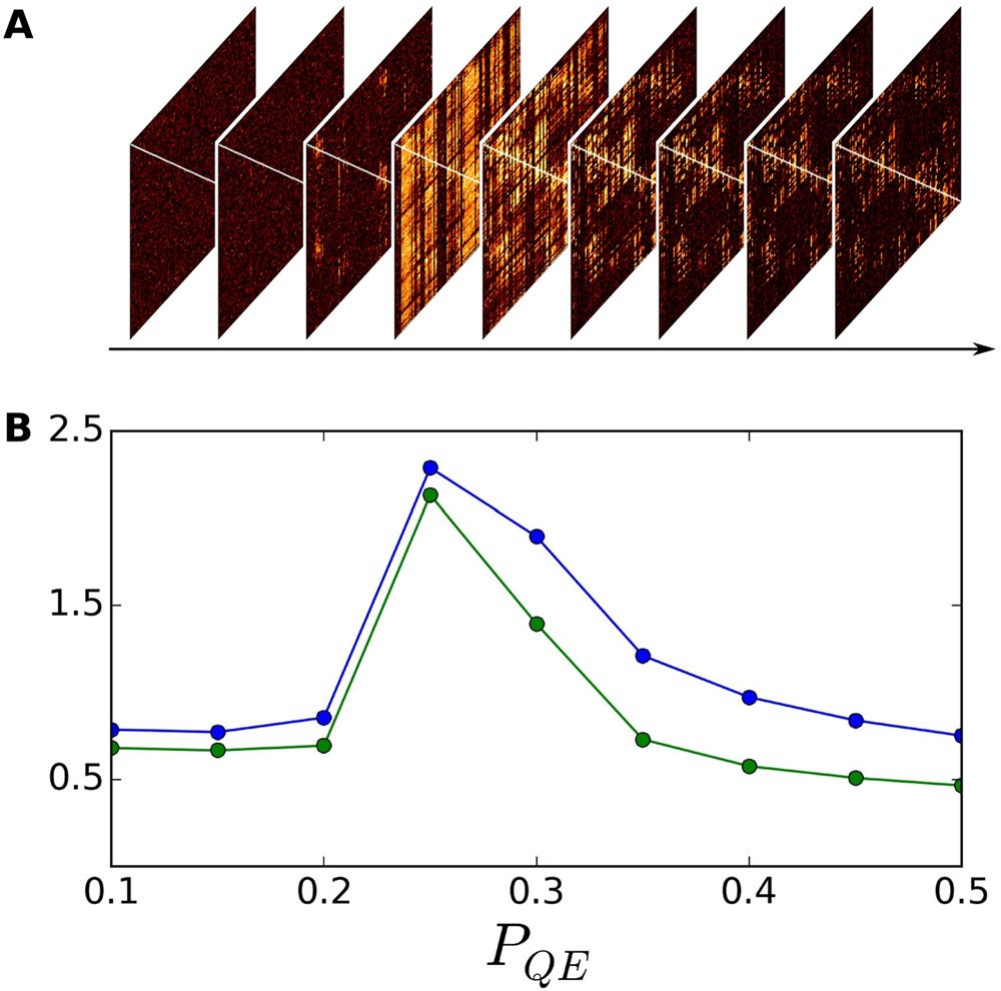
(**A**) Similarity matrices as a function of *P*_*QE*_ for *P*_*EE*_ = 0.1 and *T* = 5.2. For each similarity matrix, row indexes correspond to seeder nodes and column indexes correspond to output nodes. Then, the element *i*,*j* of a similarity matrix denotes the similarity between the input signal fed into node *i* and outputted at node *j*. The color-map represents the value of the similarity, with hot colors indicating high similarity and dark colors indicating low similarity. (**B**) Mean and median similarity values (blue and green curve respectively) as a function of *P*_*QE*_ corresponding to the similarity matrices showed in (**A**). Mean and median similarity values peak when *P*_*QE*_ is close to 0.25 (corresponding to the brightest matrix in (**A**)); for low and high values of *P*_*QE*_ the average similarity decreases.

**Figure 2:**
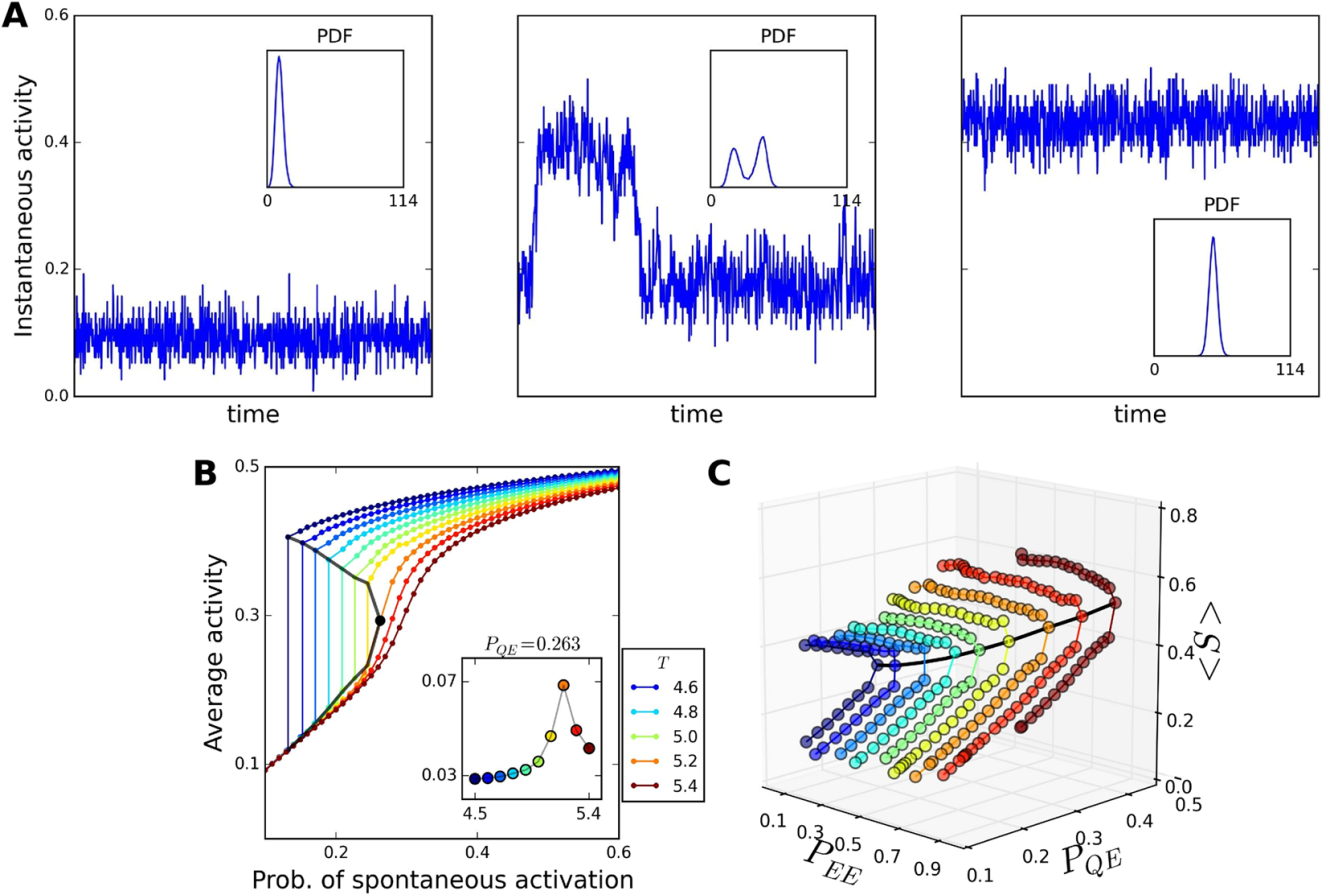
(**A**) Time series of the density of active nodes and as inset the PDF of the number of active nodes for a system with *P*_*EE*_ = 0.1,*T* = 4.8 and *P*_*QE*_ = 0.1,0.19 and 0.3. The left panel shows that the system is in the low activity level, this is confirmed when looking at the unimodal PDF of the number of active nodes that shows a maximum around 10. The middle panel shows that the system is jumping around two different levels of activity; the PDF of the number of active nodes is bimodal with two peaks around 20 and 45. The right panel shows that the system is around the high activity level around 50 number of active nodes, as it is shown in the inset with the unimodal PDF. (**B**) Average activity for *P*_*EE*_ = 0.1 and different values of *T* (each color represent a different value of *T*) as a function of *P*_*QE*_. <*S*> will exhibit a transition from a low activity level to a high activity level as a function of *P*_*QE*_. The black curve represents the coexistence curve, inside this region the two phases of the activity will coexist. The curves outside the coexistence curve increased the average activity with *P*_*QE*_ and never experienced the coexistence of two levels of activity. Inset: standard deviation of the activity for systems with *P*_*QE*_ = 0.263 and *T* from 4.5 to 5.4. It can be seen that the maximum fluctuations occur when the system is in the critical point. (**C**) Coexistence curves for values of *P*_*EE*_ from 0.1 to 0.9 (each color represent a different value of *P*_*EE*_). The system will reach higher activity levels as we increase the value of *P*_*EE*_. If the system is placed over this region, the two levels of activity will coexist. As there is one critical point for each curve, there will be a set of parameters that tune the system into a critical state (black curve).

We evaluated the similarity measure between a seeder node and all other nodes in the network, varying the three parameters of the model (*P*_*EE*_, *P*_*QE*_, *T*) as follows: we first fixed the value of *P*_*EE*_ and *T* and then vary *P*_*QE*_. Figure 1A shows pairwise similarity matrices as a function of *P*_*QE*_ for fixed values of *P*_*EE*_ and *T* (comparison of our results with a null model can be found in Fig. 2 at the Supplementary Material section). The *i* ^*th*^ row of these matrices corresponds to an instance in which the input signal was introduced through node *i* and all other nodes act as output nodes for which we measure the *similarity* between their activity time-series and the signal introduced at node *i*. We note that for these experiments we use the same input signal for all nodes.

Our findings show that, for a fixed configuration of *P*_*EE*_ and an appropriate value for *T*, there is a non-zero value of *P*_*QE*_ for which the mean and median similarity between input signals and output signals is maximized. In other words, our results confirm that there is a level of noise that enhances the transmission of the signal through the system; the level of noise that enhances signal transmission varies depending on the configuration of the model: the higher the probability of nodes remaining active (*P*_*EE*_), the larger the amount of noise needed in order to enhance the transmission of an input signal through the system. Figure 1B shows the mean and median similarity values as a function of *P*_*QE*_ for a case in which the model parameters are *P*_*EE*_ = 0.1 and *T* = 5.2 and the similarity peaks at a value of *P*_*QE*_ between 0.2 and 0.3. Table 1 shows the maximum mean and median similarity values found for different values of the parameter *P*_*EE*_.

**Table 1:** For each value of *P*_*EE*_ columns 2 and 3 show the corresponding values of *P*_*QE*_ and *T* where the maximum mean and median of the similarity matrices occur (exploring the matrices with a resolution of 0.05 in the values of *P*_*QE*_).

*P*_*EE*_	*P_{QE}*	*T*	*P_{QE}*	*T*
0.1	0.25	5.2	0.263	5.2
0.2	0.25	5.31	0.261	5.31
0.3	0.3	5.6	0.281	5.6
0.4	0.3	5.9	0.303	5.9
0.5	0.3	6.2	0.312	6.2
0.6	0.35	6.6	0.331	6.6
0.7	0.35	7.2	0.369	7.2
0.8	0.4	7.8	0.394	7.8
0.9	0.45	8.5	0.419	8.5

Columns 4 and 5 show the values of *P*_*QE*_ and *T* at the critical point (exploring the parameter space with a resolution of 0.001 in the values of *P*_*QE*_).

We performed the same analysis illustrated in Fig. 1 for a random network (Fig. 2 in the Supplementary Material section) with the same number of nodes as the empirical connectome; we generated the random network by sampling node degrees from a Gaussian distribution with mean 20.92 and standard deviation 7.01 - these values are obtained from evaluating mean and standard deviation on the empirical network -, and by sampling connection weights from another Gaussian distribution with mean 0.5 and standard deviation 0.12 - these parameters are also obtained from the empirical network. The results show that SR is also present in the randomly wired system, where the noise that maximizes the similarity is around 0.25 (when *P*_*EE*_ = 0.1 and *T* = 4.3) and, as we discuss below, coincides with the critical point. Thus this effect is robust and is not exclusive of the connectome architecture.

### Parameter space and criticality

To gain a deeper understanding of the dynamics of our system and understand why the similarity increases for an intermediate value of *P*_*QE*_, we studied the behavior of the system throughout the space of parameters in the absence of an input signal. We measured the instantaneous density of active nodes $S(t)=\frac{1}{N}\sum _{i=0}^{N}{s}_{i}(t)$, and set the time average of *S*(*t*) as the order parameter –specifically $\langle S\rangle =\frac{1}{L}\sum _{t={t}_{0}}^{{t}_{0}+L}S(t)$ (where *t*_0_ is a transient time in which the system equilibrates); varying the parameters *P*_*QE*_, *P*_*EE*_ and *T*. When looking at the instantaneous density of active nodes, for a fixed value of *P*_*EE*_, we found that the system changes discontinuously from a low to a high activity level for some values of *T* as we increase the value of *P*_*QE*_ (Fig. 2A). Hence, there is a value of *P*_*QE*_ for which the activity of the system “jumps” between the low and high activity phases (middle panel of Fig. 2A). In order to determine the average activity 〈*S*〉 taking place at a fixed value of *P*_*QE*_, we fit a Gaussian distribution to the probability distribution function (PDF) of *S*(*t*) and take the mean of the distribution as the value of 〈*S*〉. For the bimodal case, when the system is unstable and jumping between a high and low activity phase we fit one Gaussian distribution to each mode of the PDF of *S*(*t*); each of the means of the two Gaussian distributions represent the average activity 〈*S*〉 of the low and high activity phases, respectively; and the standard deviation of the activity is then computed as the mean of the standard deviations of each of the two Gaussian distributions.

Figure 2B shows the behavior of the system for a fixed value of *P*_*EE*_ and varying values of *T* and *P*_*QE*_. For a large value of *T* the average activity is a monotonically increasing function of *P*_*QE*_. For small values of both *T* and *P*_*QE*_, the system’s average activity is initially low. There is a range of *P*_*QE*_ and *T* values for which the activity of the system will jump between the high and low activity phases, these values will define the coexistence curve.

For a fixed *P*_*EE*_, the corresponding *coexistence curve* is defined by all the points in the parameter space such that the PDF of *S*(*t*) is bimodal. The black line in Fig. 2B shows the coexistence curve for *P*_*EE*_ = 0.1. Notice that, within the coexistence curve, as T increases, the difference between the average high and low activity phases decreases, which is the result of the two modes of the PDF of *S*(*t*) approaching each other. Eventually, the two modes converge onto a single mode PDF and the average activity of the system fluctuates around a single value (black marker in Fig. 2B). Interestingly, at this point, the standard deviation of the system’s activity *S*(*t*) reaches its maximum, as shown in the inset in Fig. 2B. At this point, the system is *critical* (See Methods and Supplemental Materials sections).

For each value of *P*_*EE*_ we find the corresponding coexistence curve and the critical point within the parameter space. Because there is one coexistence curve and one critical point for each *P*_*EE*_ value, the set of coexistence curves corresponding to all values of *P*_*EE*_ delimits an unstable region where the system exhibits the high and low activity phases; and finally, the set of critical points corresponding to all values of *P*_*EE*_ defines the line where the system is critical (black line in Fig. 2C).

For the remainder of the manuscript we show results corresponding to *P*_*EE*_ = 0.1, but results are qualitatively similar for other values of *P*_*EE*_ (figure SM3 shows results for systems with *P*_*EE*_ = 0.5 and 0.9).

### The coexistence of SR and Criticality

**Figure 3:**
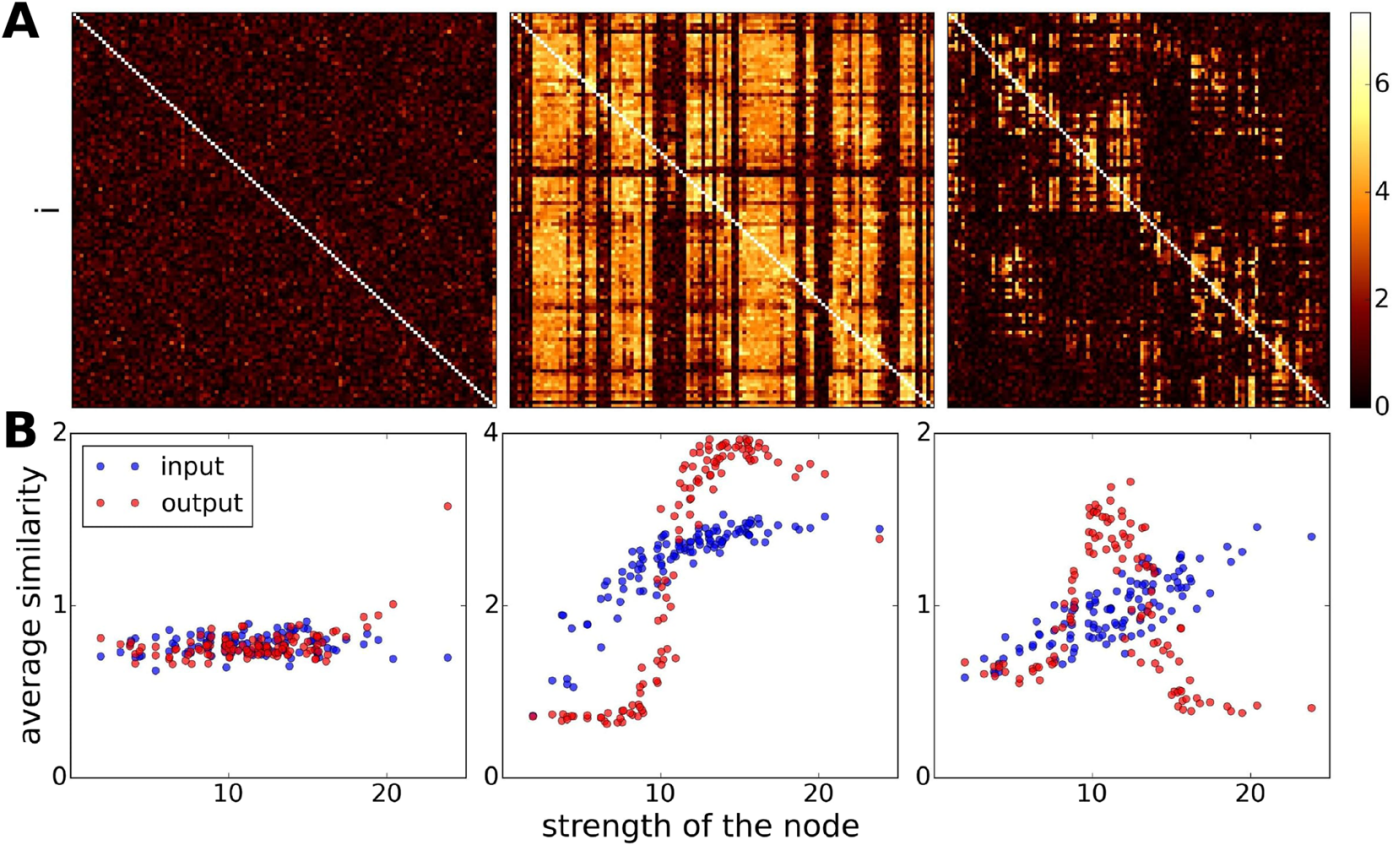
(**A**) *Similarity* matrices evaluated for three different conditions: non critical in the low activity level (*P*_*EE*_ = 0.1, *T* = 5.2 and *P*_*QE*_ = 0.15); critical point (*P*_*QE*_ = 0.263); and non critical in the high activity level (*P*_*QE*_ = 0.4). The maximum values of similarity are obtained when the system is at the critical point. (**B**) Average similarity over inputs (rows) or outputs (columns) as a function of the strength of the nodes ${w}_{i}={\sum }_{j}^{{k}_{i}}{w}_{ij}$.

Having characterized the parameter space of the system, we study the transmission of an input signal for different configurations. For these analyses, we exclude configurations corresponding to the coexistence of distinct phases because at these configurations the system can jump from one activity level to the other at different times over one realization; this generates spurious interdependencies and leads to erroneous results. Figure 3A shows examples of pairwise similarity matrices evaluated at two non-critical points (left and right panels) and a critical point (center panel).

**Figure 4:**
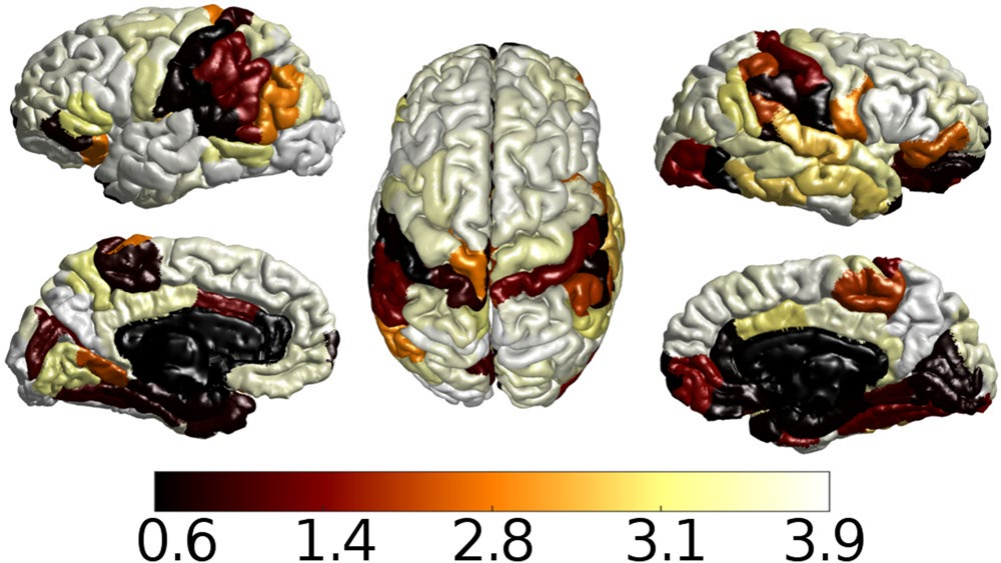
Average similarity across output nodes projected on the human cortical surface. Anatomical brain regions that suppress the input signal’s principal frequencies are: bank of the superior temporal suculus, cuneus, entorhinal cortex, frontal pole, fusiform gyrus, lateral occipital cortex, lateral orbitofrontal cortex, lingual gyrus, medial orbitofrontal cortex, paracentral lobule, parahippocampal cortex, pars orbitalis, postcentral gyrus, rostral anterior cingulate cortex, superior temporal cortex, supramarginal gyrus, temporal pole, transverse temporal cortex. The majority of these regions participate in the visual system.

Our results show that similarity values are significantly higher for most pairs of input-output nodes when the system is critical (Fig. 3A, middle panel). In other words, the parameter configurations that set the system to a critical state coincide with the parameter values at which the transmission of a signal through the network is enhanced. More specifically, the noise level, implemented by the parameter *P*_*QE*_, at which the similarity peaks in Fig. 1B, coincides with the noise level that sets the system to criticality (given appropriate values of *P*_*EE*_ and *T*; see supplementary material section Fig. 4 for different critical parameters and qualitatively similar results obtained from other measures used to assess the similarity between input and output signals).

However, the input signal is not transmitted to the entire system: we find that there is a set of “deaf” nodes whose output signal shows extremely low similarity to the input signal, regardless of what node we select to introduce the input signal. This behavior is expressed by the dark column-like patterns in the similarity matrix of the critical point. Interestingly, these nodes tend to have higher values of similarity when the system is not critical. Thus, the input signal’s principal frequencies are suppressed at these nodes when the system is critical. We also note that the column-like patterns expressed in the similarity matrices at the critical point suggest that, with a few exceptions (e.g. output-nodes within the right hemisphere frontal pole, right hemisphere medial orbitofrontal cortex, right hemisphere parahippocampal region, right hemisphere entorhinal region, right hemisphere temporal pole, left hemisphere postcentral region, left hemisphere supramarginal gyrus, and left hemisphere transversal temporal gyrus), the system’s dynamics at criticality do not vary greatly as a result of varying the input node. This is not the case when the system is not critical in which case the patterns of similarity vary depending on who the input node is, as shown by the variability across rows in the similarity matrices shown in Fig. 3A, right and left panels.

In order to gain more insight about the identity of these deaf nodes and what causes these nodes to be deaf to the input signal when the system is critical, we examined the average similarity of each output node across all input nodes (i.e. we compute an average across the columns of the similarity matrix) and the average similarity of each input node across all outputs (i.e. we compute an average over the rows of the similarity matrix).

Figure 3B shows similarity averages across inputs (blue markers) and outputs (red markers) as a function of the strength of the nodes, where strength is defined as the total sum of all connection weights of a node. We note that at the critical point (middle panel) and at the non-critical point with high average activity 〈*S*〉 (right panel) we find a clear relationship between average similarity values and node strength. Yet, the relationship between average similarities and node strength varies. At the critical point, the average similarity across output nodes resembles a step function with input-output similarity drastically increasing when the node strength exceeds a threshold value. Outside of criticality for a point with high activity (right panel) the output average similarity increases with node strength at first, but then decreases as node strength continues to increase. Average similarity across input nodes increases slowly as a function of node strength. For a non-critical point with low average activity (left panel), we find no relationship between average similarity and node strength.

Finally, Fig. 4 shows the average similarities across outputs projected on a template cortical surface, allowing us to identify the location of “deaf nodes” (indicated by dark colors). Interestingly, the dark colored cortical regions correspond to primary sensory cortical areas and the primary motor cortex of the brain, which are thought to be specialized areas that are responsible for sensory information processing and processing of motor commands, respectively. On the contrary, the lighter colored areas are also identified as the highest strength nodes or hubs which are associated with multiple, higher-order cognitive domains^40,41^ and moreover, have been shown to be crucial for the efficient integration of information^42–45^.

## Discussion

The main goal of this work was to determine whether noise can enhance the transfer of information within a simple dynamic model of the brain, and if so, to determine whether this noise corresponds to the value that tunes the system to a critical state. Our findings indicate that a noise level different from zero indeed promotes signal transmission and communication through the network, in line with what experimental evidence has shown^3,4^.

Further, we confirm that when the system is in a critical state, transmission of signals is *maximized* as evaluated by our similarity measure (we have also shown that qualitatively similar results hold when using Mutual Information and correlations). Additionally, having explored the system’s parameter space (Fig. 2C) we have found a set of parameter values at which the system is at criticality. This contrasts with previous models, which are critical at a unique point in their parameter space^28^. The multiplicity of critical points observed in our crude model may, nevertheless, be related to the brain’s capacity to adapt to different environments and/or cognitive demands. Thus, as extrinsic conditions change, the system can adjust its parameters to remain at criticality. The hypothesis that the brain operates continuously at or near a critical point has been explored previously. Tagliazzucchi *et al*.^46^, used a spatiotemporal point process over the BOLD signals recorded with fMRI, and calculated the residence time distribution of the brain at resting state. They find that the resting brain spends most of the time near the critical point^46^. Hence, if the parameters that tune the system into critical state (in our case *P*_*QE*_,*P*_*EE*_ and *T*) were time dependent, they should vary in such a way that the brain would spend more time near criticality than at other possible configurations. However, it is also conceivable that being at a critical state is energetically demanding and may not be possible for the brain to sustain such a state for prolonged periods of time^47,48^. In this situation, it is feasible that the brain “steps out” of the critical state affecting its response to external stimuli^49,50^.

We stress that even when SR could be observed at different noise levels, the critical regime appears as the best condition for the transfer of signals through the system. Measurements of similarity revealed that the values obtained in the critical regime are larger than those measured when the system is at a non-critical state (Fig. 3). Interestingly, even when the system is at a critical state, there is a set of nodes that exhibit an incapacity to communicate with the rest of the network (dark columns in the center panel of Fig. 3). These findings can be related to well known functional aspects of the different brain areas, as it turns out that the “deaf” nodes are not disconnected from the network, but rather, belong to unimodal or primary sensory processing areas such as the motor cortex^51,52^, auditory and speech areas^53,54^. These specialized or unimodal areas are known to process information in a segregated manner^55,56^ while also take part in integrated processing^50,57–60^. In agreement with this framework, our findings support the idea that integration and segregation coexist^61–63^ as the dynamics in the brain change configurations. Our results suggest that the system is wired in such a way that when it is at criticality, it facilitates integration of information within higher-order processing areas belonging to various functional sub-systems^64^, while it also promotes segregated processing within primary sensory and primary motor areas by inhibiting the spreading of information within these specialized regions. However, if the system is not at a critical state, we observe some spreading of the input signals through the unimodal areas, particularly when the system is at a high activity phase (see Fig. 3, right panel). This supports the hypothesis that these areas can indeed engage in integrative functions as suggested in^50^.

The system studied in this paper is limited by the small size of the connectome network we used (114 nodes), making it difficult to determine the exact critical point. These networks were extracted from the combination of diffusion spectrum imaging and tractography, a widely used approach for non-invasive reconstruction of human anatomical connectivity. In future work, new non-invasive technologies are likely to contribute more detailed maps of anatomical brain networks in humans. An intriguing avenue for further investigation would be to examine individual differences in signal transmission, and changes across development and lifespan.

There are many examples of previous studies that use simple discrete state excitable dynamics to model neural activity^16,28,31,46,65^, demonstrating the capacity of such simple models to display a broad range of dynamical regimes (including criticality). Interestingly, a discrete 3-state excitable dynamics model used by Haimovici *et al*.^28^ was able to reproduce resting state brain activity^34,35^ and well known coupling relationships between functional brain sub-systems known as resting state networks^29^. These results demonstrate that simple discrete excitable dynamics models are able to capture the patterns of functional connectivity that emerge as a result of neural interactions taking place through the anatomical structure but that are not trivially explained by the anatomy itself ^66^.

Given the simplicity of the model presented here, future research could aim at finding the differences in dynamical properties using the same analysis over networks extracted from diseased brains. If the transmission of information in damaged networks is different, the noise effect described in our work could be explored as a way to improve neural communication. Another goal could be to study the cooperative and competitive effects in the spreading of a signal through neural networks^44^. It would also be interesting to study how these effects change according to the seeder nodes. These and other extensions of the present study could assist to our understanding of how communication processes contribute to various aspects of brain function.

## Methods

### Data

**Figure 5:**
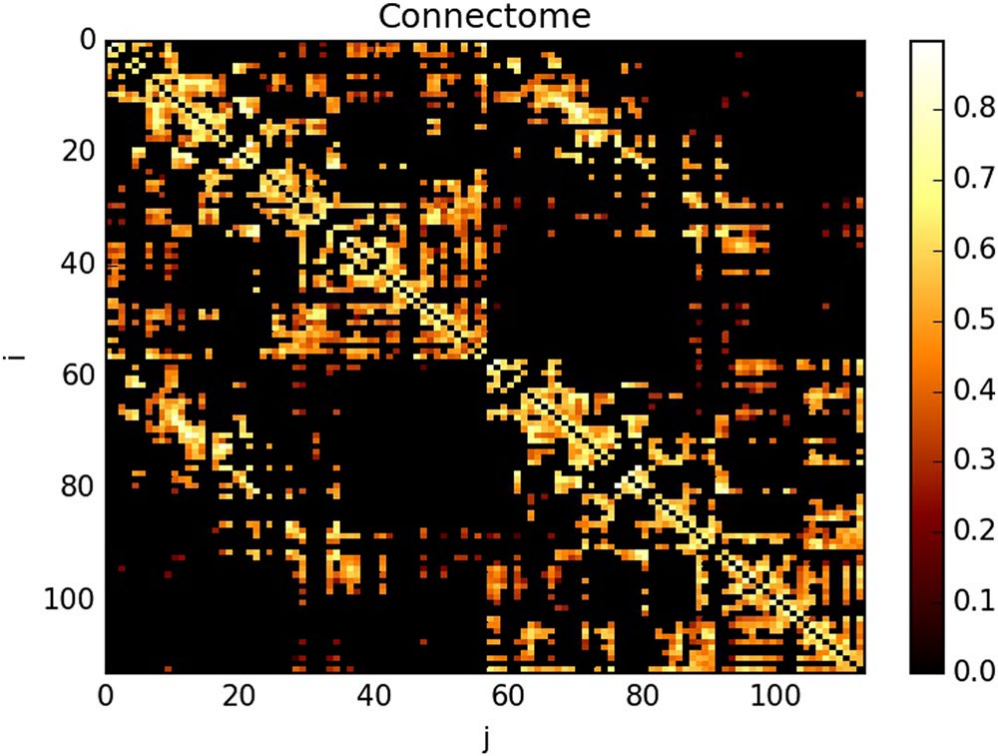
Representation of the adjacency matrix of the connectome for a network of 114 nodes. The colors in the matrix corresponded to the value of the connection between pairs of nodes. Nodes from 0 to 57 were located at the right hemisphere, whereas nodes from 58 to 114 were at the left hemisphere^27,37^.

Informed written consent in accordance with the Institutional guidelines (protocol approved by the Ethics Committee of Clinical Research of the Faculty of Biology and Medicine, University of Lausanne, Switzerland) was obtained for all subjects. Forty healthy subjects (16 females; 25.3 ± 4.9 years old) underwent an MRI session on a 3 T Siemens Trio scanner with a 32-channel head coil. Magnetization prepared rapid acquisition with gradient echo (MPRAGE) sequence was 1-mm in-plane resolution and 1.2-mm slice thickness. DSI sequence included 128 diffusion weighted volumes +1 reference b_0 volume, maximum b-value 8,000 *s*/*mm* ^2^, and 2.2 × 2.2 × 3.0 mm voxel size. EPI sequence was 3.3-mm in-plane resolution and 3.3-mm slice thickness with TR 1,920 ms. DSI and MPRAGE data were processed using the Connectome Mapping Toolkit^67^. Each participant’s gray and white matter compartments were segmented from the MPRAGE volume. The gray matter volume was subdivided into 68 cortical and 15 subcortical anatomical regions, according to the Desikan-Killiany atlas^68^, defining 83 anatomical regions. These regions were hierarchically subdivided to obtain five parcellations, corresponding to five different scales^69^. The present study uses a parcellation comprising 129 regions of interest (ROI); however, here we focus on cortical structures only, discarding all subcortical regions including the bilateral thalamus, caudate, putamen, pallidum, nucleus accumbens, hippocampus, and amygdala, as well as the brainstem, resulting in 114 remaining ROI. Whole brain streamline tractography was performed on reconstructed DSI data^70^, and connectivity matrices were estimated from the streamlines connecting each pair of cortical ROI. We quantify the connection strength between each pair of regions as a fiber density^71^ instead of fiber count. Thus, the connection weight between the pair of brain regions {*u*,*v*} captures the average number of connections per unit surface between *u* and *v*, corrected by the length of the fibers connecting such brain regions. The aim of these corrections is to control for the variability in cortical region size and the linear bias toward longer fibers introduced by the tractography algorithm. Fiber densities were used to construct subject-wise structural connectivity matrices. Finally, we construct a group connectome from all 40 individual subject connectomes following the consensus approach described in^44^, where edges that are most frequently found across all individual are selected to conform the group connectome. Following the edge-weight transformation procedure described in^37^, our average connection matrix (Fig. 5) was obtained after fiber-density edge weights were re-sampled to a Gaussian distribution.

### Parameter space

Overall, the phase diagram of our system (Fig. 2B) resembles to the liquid-gas transition described by the Van der Waals equation. In the Van der Waals theory of the liquid-gas transition, the isotherms can take different shapes on the pressure-density plane depending on the value of the temperature T. For a large value of T, the density is a continuous monotonically increasing function of the pressure. In contrast, when T is low enough, there are some values of the pressure in which the system can have two different densities. At these points the system undergoes a discontinuous phase transition from gas to liquid or vice versa. The densities at which this transition occurs delimit the coexistence curve, where liquid and gas phases can coexist at the same temperature and pressure. The end point of the coexistence curve, at which the transition becomes continuous, is the critical point of the system, and lies on the isotherm corresponding to the critical temperature^72^.

**Figure 6:**
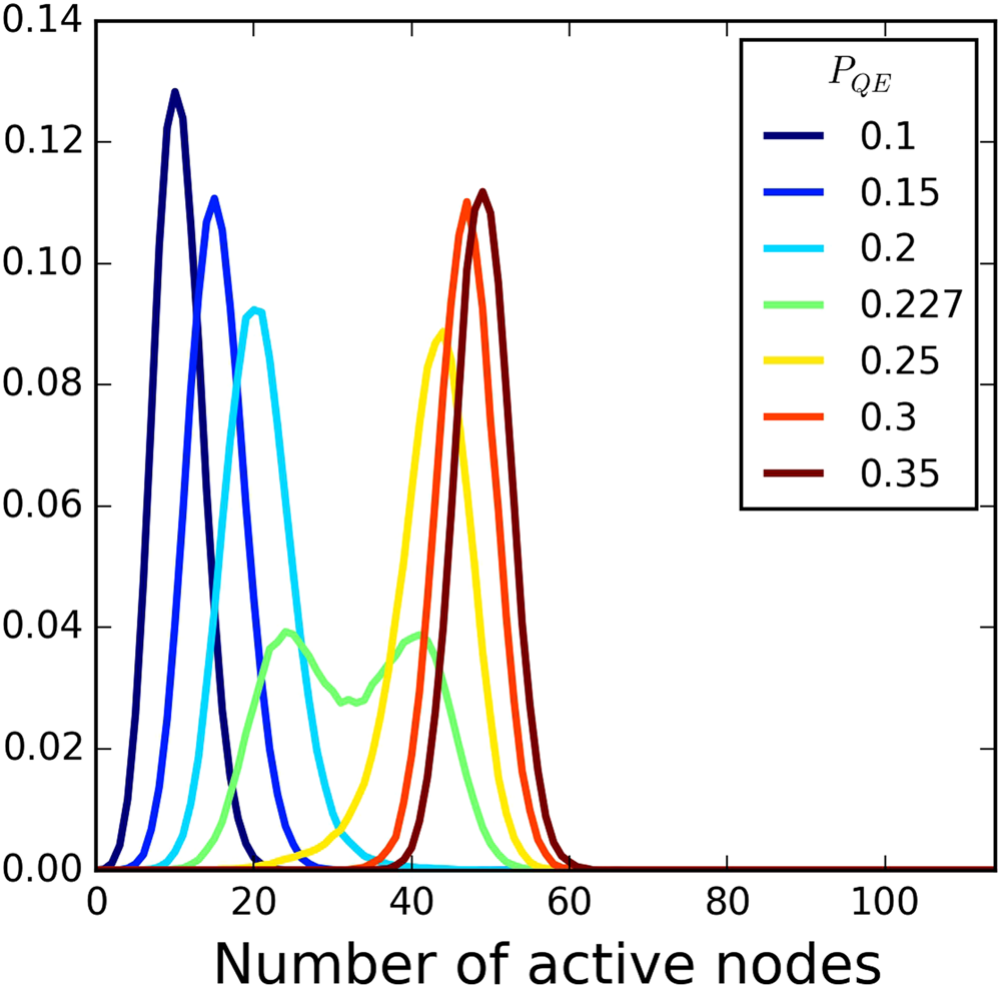
Probability distribution function for the number of active nodes for a system with *P*_*EE*_ = 0.1 and *T* = 5.0. As we increase *P*_*QE*_ the system goes from a low to a high activity level. When the probability is bimodal (*P*_*QE*_ = 0.227) the system is at the coexistence curve.

Making an analogy to the Van der Waals fluid, we constructed the phase space of our system in the following way. In order to determine the coexistence curve, we measured the distributions of values *S*(*t*) attained in each run for fixed values of *P*_*EE*_ and *T*, increasing *P*_*QE*_ until the probabilities of being in the low or high activity state were the same (when the two maximums in the distribution of the activity have the same heights, as in Fig. 6). We fit a sum of two Gaussian distributions to the PDF and the two “means” set the values of the average activity over the coexistence curve. Outside the coexistence curve, the values of the activity are simply given by the average activity value <*S*>.

**Figure 7:**
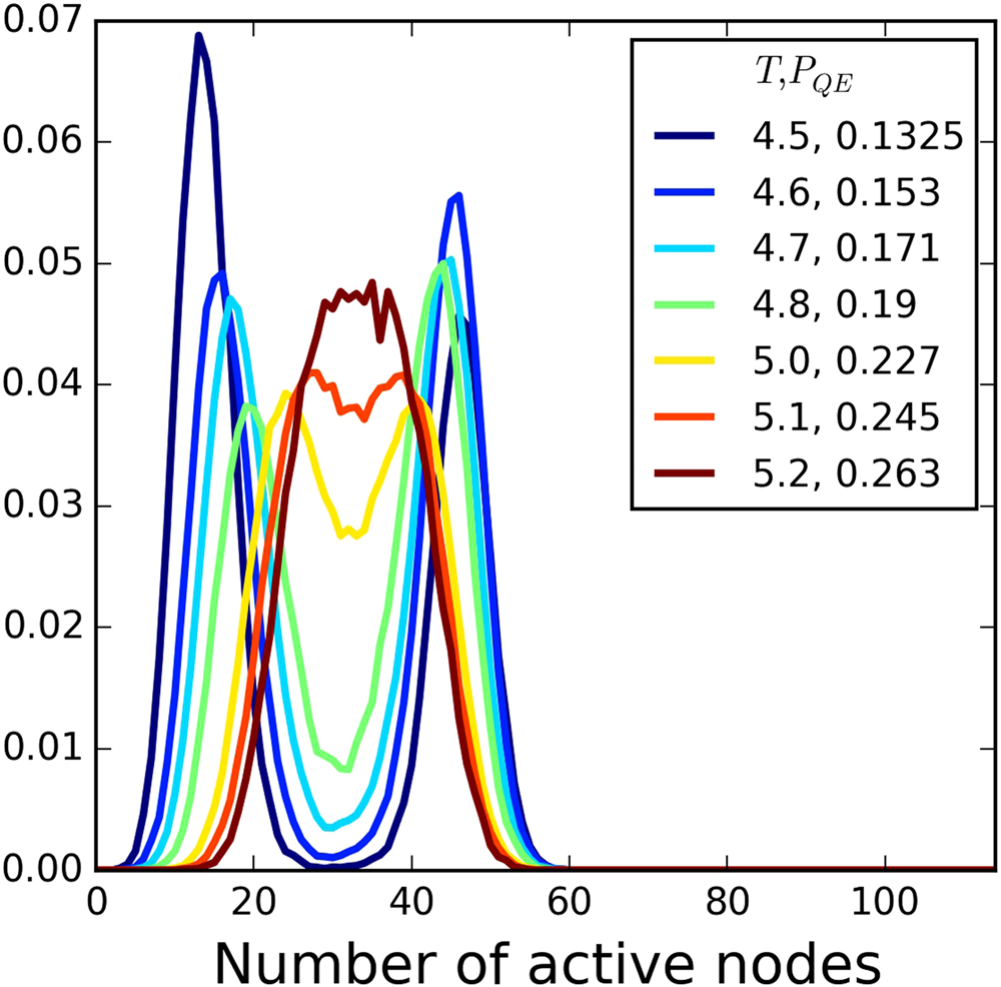
Probability distribution function for the number of active nodes for a system with *P*_*EE*_ = 0.1. As we increase *T* the difference between the low and high levels of activity decreases until they overlap at the critical value of *T*.

**Figure 8:**
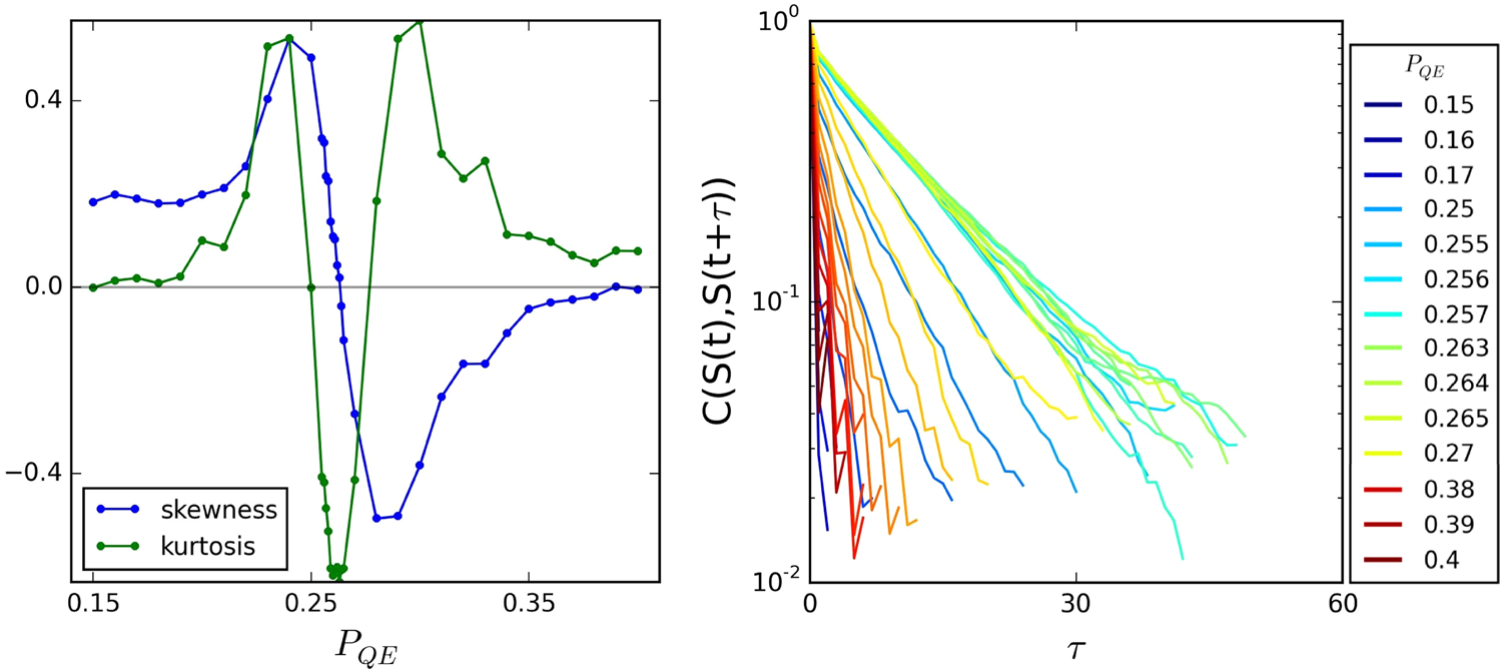
For a system with *P*_*EE*_ = 0.1 and *T* = 5.2 the skewness, kurtosis (left panel) and autocorrelation length (right panel) for different values of *P*_*QE*_. We can see that when *P*_*QE*_ is around 0.26 the kurtosis and autocorrelation length do not change too much and the skewness crosses zero, as we expect for the system when it is near criticality.

When we increase the value of *T*, the difference between the two levels of activity at coexistence will decrease (the two maximums in the distribution will get closer). Thus, for a fix value of *P*_*EE*_ we look for the *T* at which the two levels of activity overlap (Fig. 7). Once we have the critical *T*, we compute the skewness and the kurtosis for the distributions, as well as the autocorrelation time obtained from runs at different values of *P*_*QE*_. The distribution with a skewness closest to zero, a negative kurtosis and the largest autocorrelation time was chosen as critical (Fig. 8). The activity of the system at this point will be fluctuating around a single value and will have the highest standard deviation.

## Electronic supplementary material

Supplementary PDF File
