# Supplementary material for: Stochastic resonance at criticality in a network model of the human cortex
Source: Sci Rep. 2017 Oct 12;7:13020. doi: 10.1038/s41598-017-13400-5 (PMC5638949; doi:10.1038/s41598-017-13400-5)
Supplement: Supplementary file 1 — Supplementary PDF File [file 41598_2017_13400_MOESM1_ESM.pdf]

Supplementary Material for "Stochastic resonance at criticality in a network model of the human cortex"

Bertha Vázquez-Rodríguez[1,*], Andrea Avena-Koenigsberger[2], Olaf Sporns[2], Alessandra Griffa[3,4], Patric Hagmann[3,4], and Hernán Larralde[1]

[1]Universidad Nacional Autónoma de México, Instituto de Ciencias Físicas, Cuernavaca, México
[2]Indiana University, Department of Psychological and Brain Sciences, Bloomington IN, USA
[3]Lausanne University Hospital (CHUV), Department of Radiology, Lausanne, Switzerland
[4]University of Lausanne (UNIL), Lausanne, Switzerland
[*]bertha@fis.unam.mx

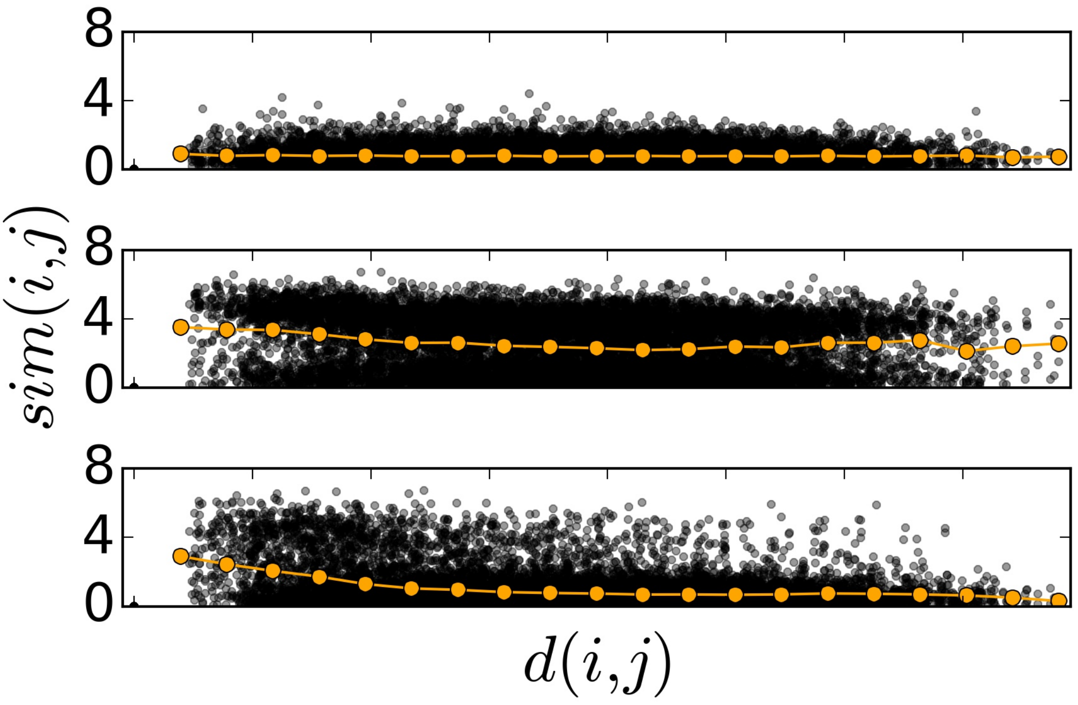

Figure S 1: **Similarity as a function of the euclidean distance.** Similarity between pairs as a function of the euclidean distance between nodes for systems with $P_{EE} = 0.1, T = 5.2$ and $P_{QE} = 0.15, 0.263$ and 0.4 (low activity level, critical and high activity level respectively. The similarity does not depend on the distance between nodes, as can be seen from the scatter plot of the similarity as a function of the euclidean distance for a system at the critical point and systems out of criticality.

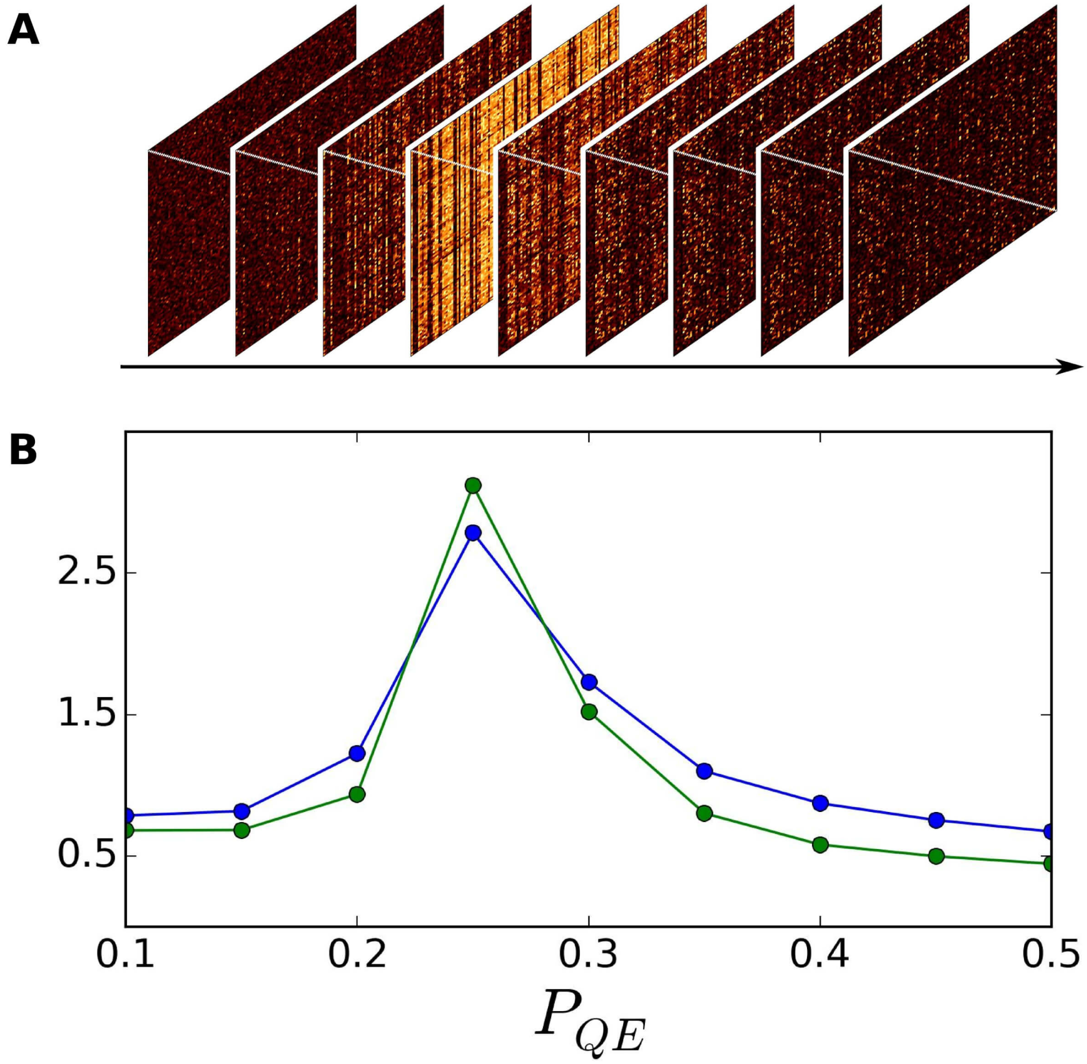

Figure S 2: **Stochastic resonance.**(A) Similarity matrices for a random network with a Gaussian distribution of the number of inputs (mean 20.92 and standard deviation 7.01), and a Gaussian distribution of the weights (mean 0.5 and standard deviation 0.12). The noise that maximize the similarity is around 0.25, hence the presence of SR is no dependent on the network structure. (B) The mean and median of the similarity matrices show a peak for an intermediate value of P_{QE} which is representative of SR.

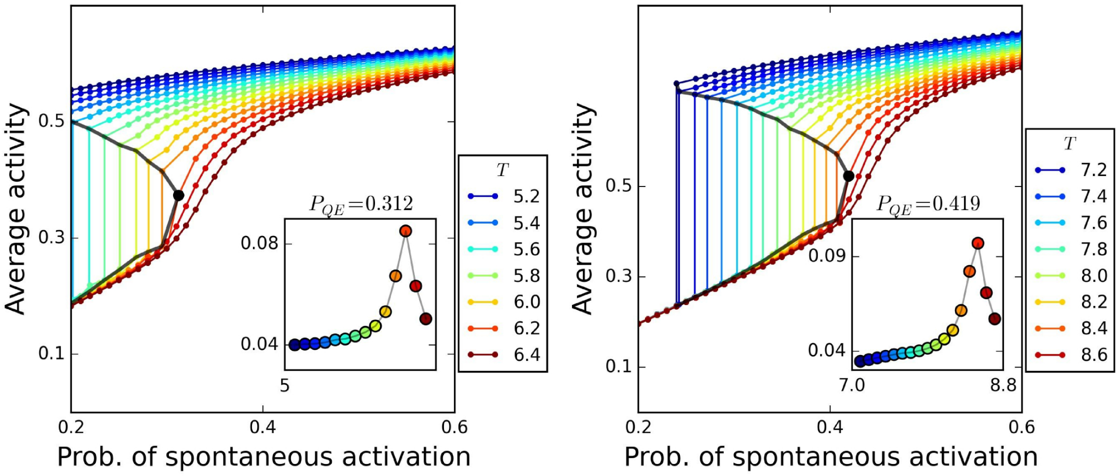

Figure S 3: **Parameter space for two different levels of $P_{EE} = 0.5$ and 0.9.** The phase space is qualitatively similar to the one shown at the results section for $P_{EE} = 0.1$. The average activity as a function of T and P_{QE} for different values of $P_{EE} = 0.5$ (left), 0.9 (right). The black dot represent the corresponding critical point for that level of P_{EE}. Inset: standard deviation of the average activity for the corresponding critical value of P_{QE} and a set of different values of T. The parameter space is equivalent, no matter which is the value of P_EE. The differences are that the high activity level is larger as we increase P_{EE}, and that the critical point appear at higher values of P_{QE} and T.

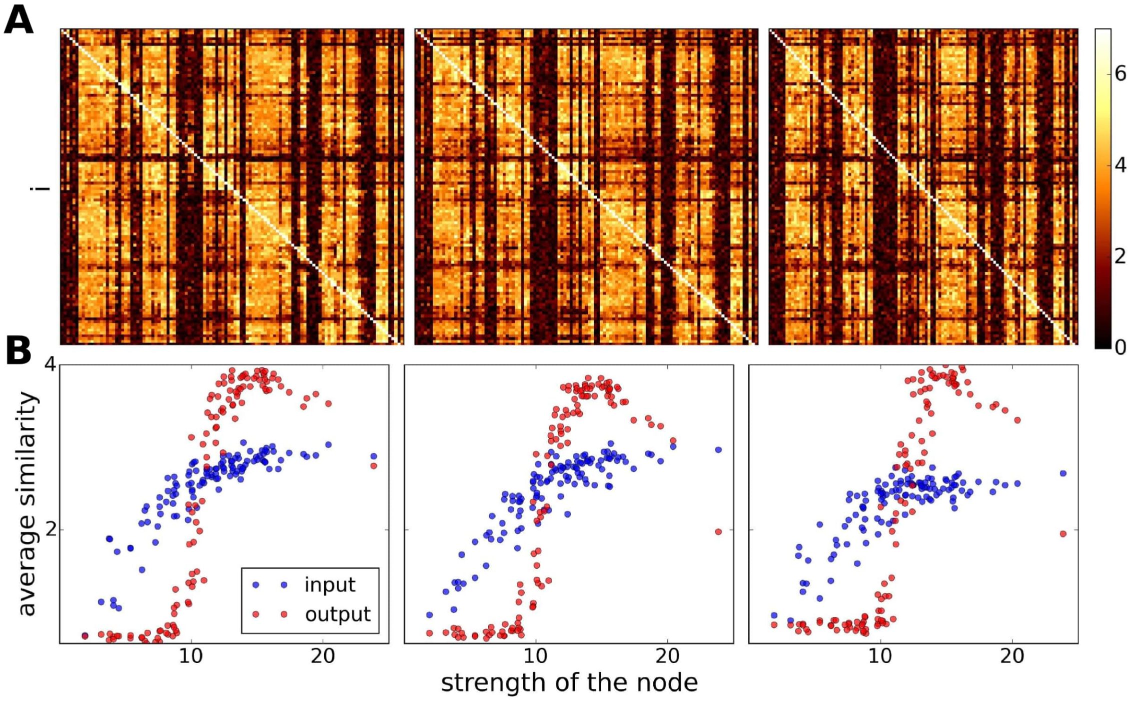

Figure S 4: **Similarity matrices for different critical points.** (A)*Similarity* matrices evaluated for the critical parameters corresponding to a system with $P_{EE} = 0.1, 0.5$ and 0.9. (B)Average similarity over inputs (blue) or outputs (red) as a function of the strength of the nodes.

Information measurements

Other ways to quantify the transmission of information between nodes would be to measure mutual information and cross correlation as a function of the delay τ. These measurements assess how the statistics of the activity at one node are related to the activity at another node. The mutual information between nodes i and j is defined as [1]:

$$MI(s_i, s_j, \tau) = \sum_{a=0,1} \sum_{b=0,1} P(s_i(t) = a, s_j(t+\tau) = b) \times \log \left(\frac{P(s_i(t) = a, s_j(t+\tau) = b)}{P(s_i(t) = a) P(s_j(t+\tau) = b)} \right),$$

where $P(x)$ is the probability of x, and $P(x, y)$ is the joint probability for x and y.

Cross correlation with delay τ was computed as:

$$C(s_i, s_j, \tau) = \left| \frac{1}{(L-\tau)\sigma_i \sigma_j} \sum_{t=0}^{L-\tau} (s_i(t) - \mu_i)(s_j(t+\tau) - \mu_j) \right|,$$

where μ_i is the average value of the activity $s_i(t)$ at node i and σ_i is the standard deviations [1].

We calculated the mutual information (fig. 8) and correlation (fig. 6) in this way for all pair of nodes and the same set of parameters as for the similarity ($P_{EE} = 0.1, T = 5.2, P_{QE} = 0.15, 0.263$ and 0.4).

To determine the value of spurious correlations, we also computed $C(s_i, s_j, \tau)$ using s_i and s_j taken from different realizations with the same parameter values. Since these signals are perforce independent, the maximum value reached sets a threshold below which correlations in the systems cannot be distinguished from spurious correlation.

When we compute the actual correlation between nodes from the same realization, we define the correlation time as the first time the value of the correlation falls below the threshold established above (fig. 5). We record the maximum correlation and mutual information (figures 6, 8) between the pair of nodes and the time during which the nodes were correlated or sharing information (figures 7, 9).

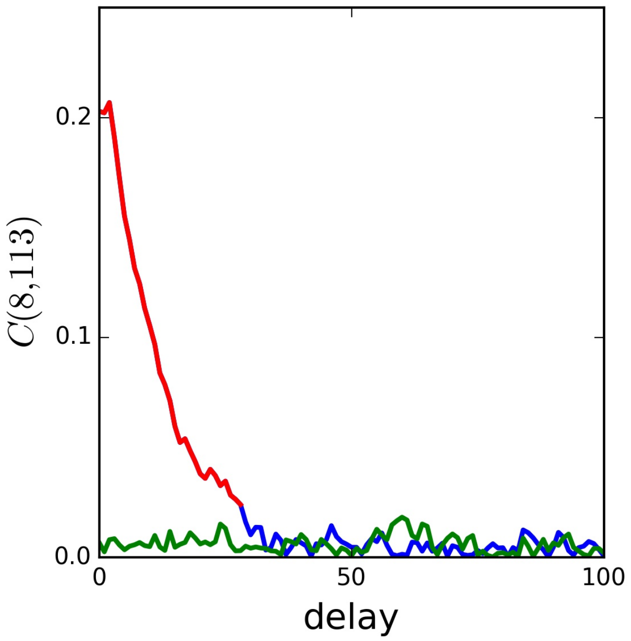

Figure S 5: **Distinction between real and spurious correlations.** The correlation between a pair of nodes from different realizations (green curve) should be zero due to the independence, so it establish a threshold to consider a correlation as spurious. The time the nodes were correlated (red dots) is determined when the correlation obtained for nodes from the same realization (blue curve) is lower than the threshold.

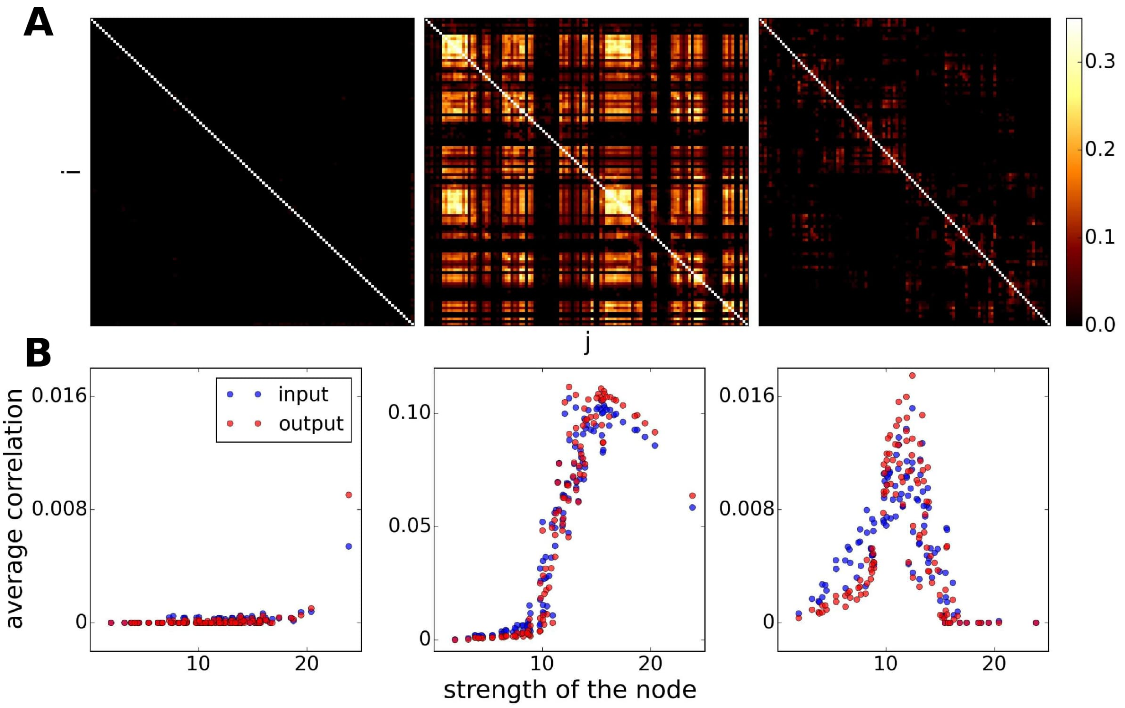

Figure S 6: **Correlation matrices for three different conditions.** (A)Maximum correlation between pairs of nodes with $P_{EE} = 0.1, T = 5.2$ and $P_{QE} = 0.15(non-critical low activity level)$, 0.263 (critical) and 0.4 (non-critical high activity level). (B)Average maximum correlation over inputs (blue) or outputs (red) as a function of the strength of the nodes.

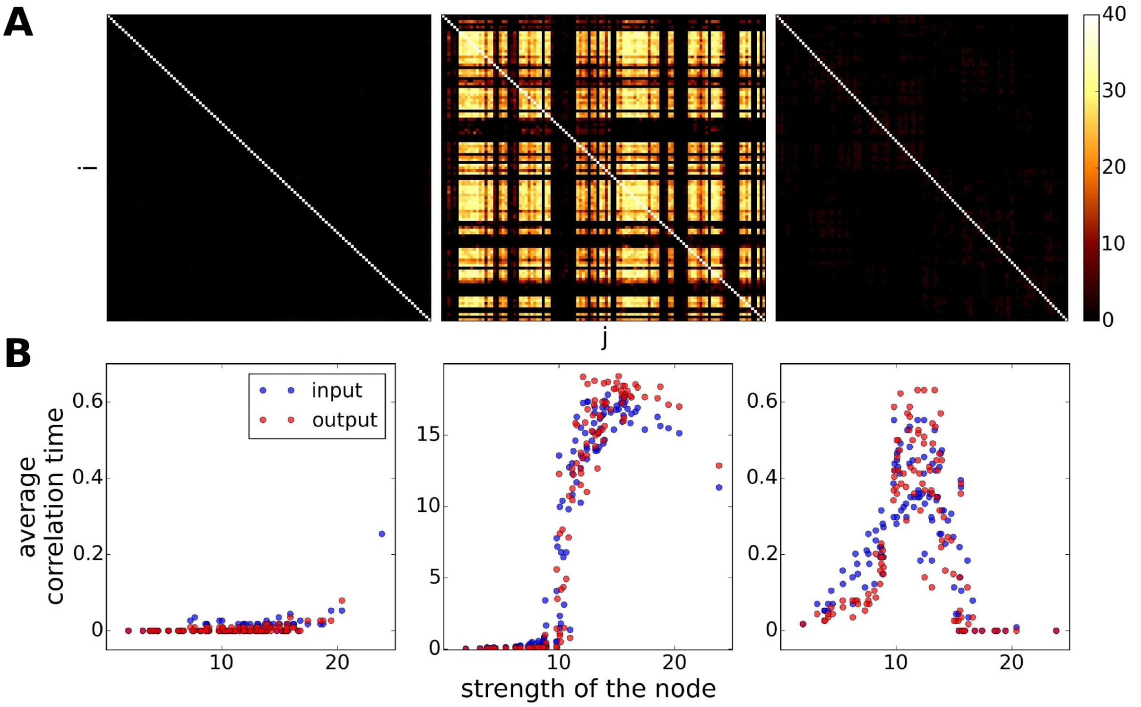

Figure S 7: **Time interval over which the nodes are correlated.**(A)Matrices of the time interval over which the nodes are correlated with $P_{EE} = 0.1, T = 5.2$ and $P_{QE} = 0.15(non - criticallowactivitylevel), 0.263$ (critical) and 0.4 (non-critical high activity level). (B)Average correlation time over inputs (blue) or outputs (red) as a function of the strength of the nodes.

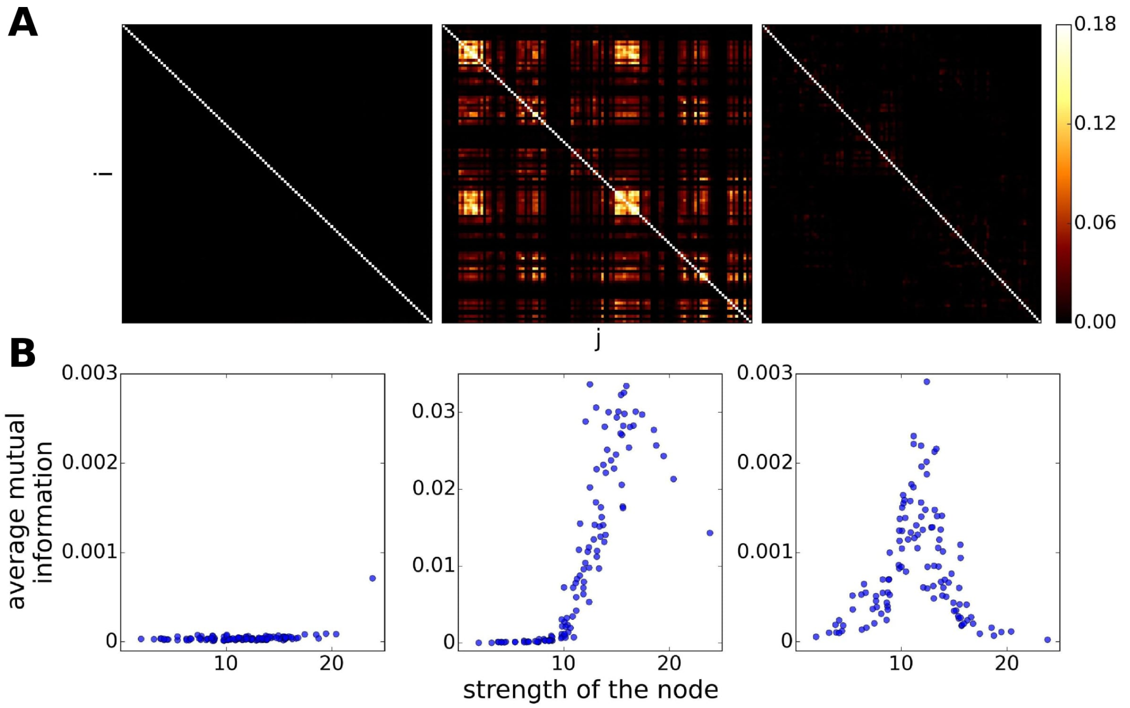

Figure S 8: **Mutual information for three different conditions.**(A)Maximum mutual information between pairs of nodes with $P_{EE} = 0.1, T = 5.2$ and $P_{QE} = 0.15(non - criticallowactivitylevel), 0.263$ (critical) and 0.4 (non-critical high activity level). (B)Average maximum mutual information over inputs as a function of the strength of the nodes.

References

[1] Peter Dayan and Laurence F Abbott. *Theoretical neuroscience*, volume 10. Cambridge, MA: MIT Press, 2001.

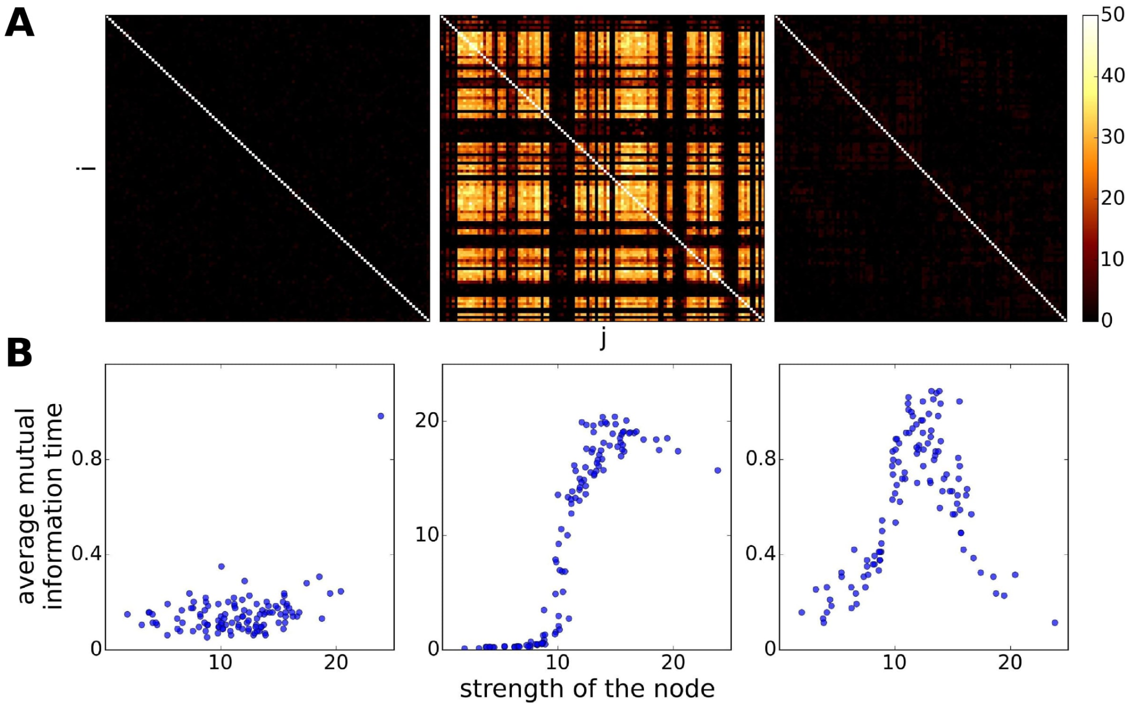

Figure S 9: **Time interval over which the nodes have a non spurious mutual information.** (A)Matrices of time interval over which the nodes have a mutual information larger than the one obtained for nodes from different realizations. For a system with $P_{EE} = 0.1, T = 5.2$ and $P_{QE} = 0.15 (non - criticallowactivitylevel), 0.263$ (critical) and 0.4 (non-critical high activity level). (B)Average mutual information time over inputs as a function of the strength of the nodes.
